# Novel biodegradable magnesium alloy clips compared with titanium clips for hepatectomy in a rat model

**DOI:** 10.1186/s12893-019-0600-y

**Published:** 2019-09-09

**Authors:** Takeshi Urade, Toshihiko Yoshida, Naoko Ikeo, Kosuke Naka, Masahiro Kido, Hirochika Toyama, Kimihiko Ueno, Motofumi Tanaka, Toshiji Mukai, Takumi Fukumoto

**Affiliations:** 1 Department of Surgery, Division of Hepato-Biliary-Pancreatic Surgery, Kobe University Graduate School of Medicine, 7-5-2 Kusunoki-cho, Chuo-ku, Kobe, 650-0017 Japan; 2 Department of Mechanical Engineering, Kobe University, Kobe, Japan

**Keywords:** Biodegradable clip, Absorbable clip, Magnesium clip, Metal clip, Hepatectomy, Rat

## Abstract

**Background:**

The use of surgical metal clips is crucial for ligating vessels in various operations. The currently available metal clips have several drawbacks; they are permanent and interfere with imaging techniques such as computed tomography (CT) or magnetic resonance (MR) imaging and carry the potential risk of endo-clip migration. We recently developed a novel magnesium (Mg) alloy for biodegradable clips that reduces artifacts on CT imaging. This study aimed to examine the tolerance, biodegradability, and biocompatibility of the Mg alloy clips compared with those of standard titanium (Ti) clips in hepatectomy.

**Methods:**

Thirty Wistar rats were divided into two groups based on the clip used (groups A and B). The vascular pedicle, including hepatic artery, portal vein, bile duct, and hepatic vein of the left lateral lobe, was ligated with the Ti clip in group A or the Mg alloy clip in group B, and then the left lateral lobe was removed. The rats were sacrificed at 1, 4, 12, 24, and 36 weeks after surgery. Clinical and histological evaluations were performed. Absorption rate was calculated by measuring the clip volume.

**Results:**

Although the Mg alloy clips showed biodegradability over time, there were no significant differences in the serum concentration of Mg between the two groups. The remaining volume ratio of Mg alloy clips was 95.5, 94.3, 80.0, 36.2, and 16.7% at 1, 4, 12, 24, and 36 weeks, respectively. No side effects occurred. Most of the microscopic changes were similar in both groups.

**Conclusions:**

The new biodegradable Mg alloy clips are safe and feasible in vessel ligation for hepatectomy in a rat model and reduce artifacts in CT imaging compared with the standard Ti clips.

## Background

The use of surgical metal clips is crucial for ligating vessels in various operations. The currently available metal clips are made of pure titanium (Ti) and Ti alloys, which are strong and ductile enough to occlude vessels in soft tissues using forceps. However, Ti clips have several drawbacks; they form metallic artifacts, which hamper the image quality in computed tomography (CT) evaluations, and they permanently remain in the human body, sometimes resulting in adhesion or endo-clip penetration and migration. [1–3] Some patients develop allergic reactions to Ti ions. [4] Absorbable polymer clips were developed in the early 1990s, [5] but the use of polymer clips is limited due to their thickness and weakness of clipping force.

Recently, magnesium (Mg) alloys have attracted considerable attention because of their excellent biocompatibility and biodegradability. Biodegradable instruments made of Mg alloys are used in bone screws, orthopedic implants, and dental implants used for oral maxillofacial surgery. [6–10] However, the physical properties of the Mg alloys necessary for use as operative clips differ from those of other instruments. Besides suitable biodegradability, it requires sufficient ductility and mechanical strength for enabling tissue ligation. Considering this, we developed a novel metal clip made of Mg alloys, which has enough strength for ligation, biodegradability, and reduces artifacts on CT imaging. [11, 12]

In liver surgery, hepatic transection is a key determinant of intraoperative blood loss and postoperative complications. During hepatic transection, surgical metal clips are frequently used to ligate the biliary and vascular structures to reduce surgery time. [13] In a previous study, we evaluated the feasibility of our Mg alloy clip in a canine cholecystectomy model. [12] However, we have not yet evaluated the influence of the Mg alloy clip on the vascular structure. The aims of this study were to confirm the ability of the Mg alloy clip for vessel sealing, to evaluate the serum concentration of Mg, and to check the degradation behavior when we used the Mg alloy clips for vessel ligation in hepatectomy. A rat model was used in the present study because numerous previous studies have already established the hepatectomy method and it is easy to perform CT scan repeatedly to evaluate the degradation of the clips.

## Methods

This study was conducted in accordance with the Kobe University Animal Experimentation Regulation and was approved by the Institutional Animal Care and Use Committee (permission number: P141203).

### Materials

**Fig. 1:**
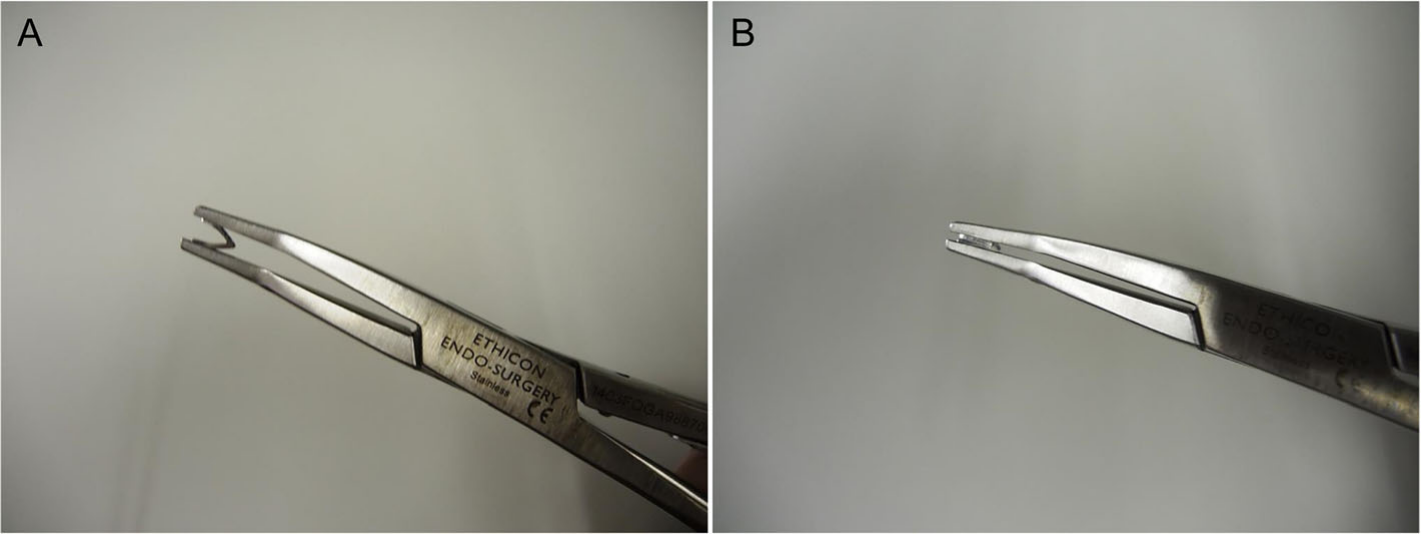
Photograph of a magnesium alloy clip. **a** A magnesium alloy clip mounted on the clip applier. **b** A closed magnesium alloy clip

The manufacturing method of the new Mg alloy clip has been described in our previous study. [11] This Mg alloy consists of 99.8% Mg, 0.2% zinc (Zn), and 0.1% calcium (Ca); it has sufficient mechanical strength and ductility for enabling tissue ligation by controlling its microstructure. A clip with a cross-section of 0.7 × 0.7 mm^2^ and a side 6 mm long was fabricated using hot extrusion. The clip was machined from the bar followed by an acid wash. The acid used in this study was 5% Nital solution (5% nitric acid + 95% ethanol). The weight after the acid wash of the clip was unified to be approximately 11 mg. A Ti clip (LIGACLIP Extra Ligating Clip, Medium, Ethicon Endo-Surgery, LLC, Cincinnati, OH) was used as a control. The clip applier (Multi-Patient Single-Clip Applier, Ethicon Endo-Surgery, LLC) was used to apply the Ti and Mg alloy clips (Fig. 1a and b).

### Animal model and surgical procedure

**Fig. 2:**
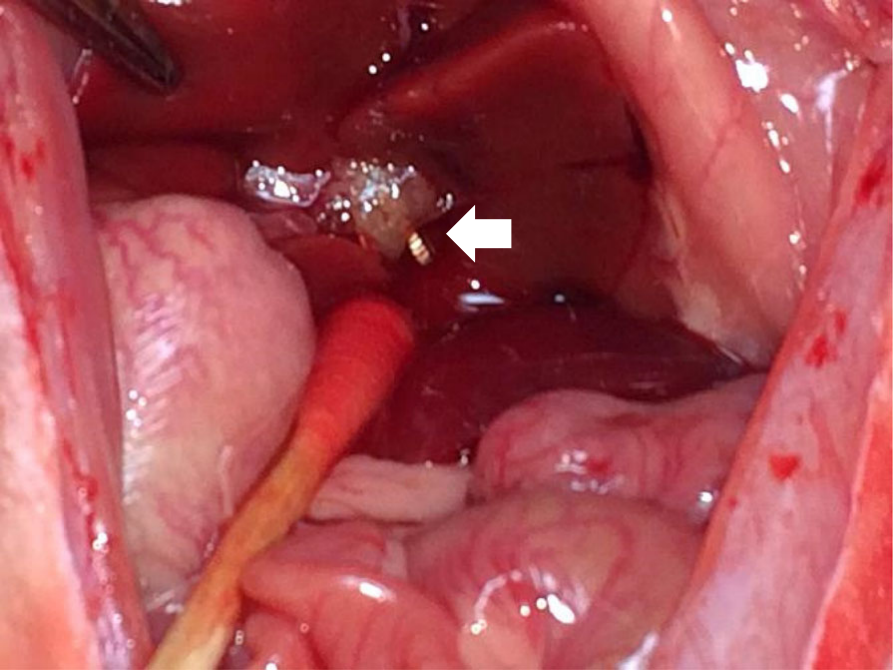
Photograph of the operative field after hepatectomy. The left lateral lobe was removed using the Mg alloy clip (white arrow)

Thirty male healthy Wistar rats (8 weeks old; weight, 310–380 g) were divided into two groups: group A was treated with Mg alloy clips (*n* = 15), whereas group B was treated with Ti clips (n = 15). The rats were supplied by Charles River Laboratories Japan, Inc. (Yokohama, Japan). Two rats were placed inside each cage in a temperature-controlled room, with food and water provided ad libitum under a 12-h light/dark diurnal cycle. Operations were conducted under general anesthesia with isoflurane. The animals were placed in a supine position, and laparotomy was performed with a midline abdominal incision. After the median lobe was retracted to the cranial side and the caudate lobe to the caudal side, the vascular pedicle, including hepatic artery, portal vein, bile duct, and hepatic vein of the left lateral lobe, was encircled. Lastly, the pedicle was ligated using the Ti or Mg alloy clip, and the left lateral lobe was removed (Fig. 2). After complete hemostasis was confirmed, the abdominal wall was closed. Three rats in each group were humanely euthanized with exsanguination under deep anesthesia in the laboratory, followed by extraction of the clips at 1, 4, 12, 24, or 36 weeks after surgery.

### Blood chemistry

Blood biochemical testing was conducted to evaluate concentrations of aspartate aminotransferase (AST), alanine aminotransferase (ALT), creatinine (Cre), calcium (Ca), and Mg. Blood samples were taken from the caudal vein preoperatively and at 1, 4, 12, and 24 weeks postoperatively in the 24-week observation group rats (group A: *n* = 3, group B: *n* = 3) and were measured by ORIENTAL YEAST Co., Ltd. (Tokyo, Japan).

### Micro-CT imaging

Micro-CT imaging using an in vivo micro-CT scanning machine (R_mCT2, Rigaku, Japan) was also performed under general anesthesia with isoflurane. The reaction of the intra-abdominal implanted clips was evaluated just after surgery and at 1, 4, 12, 24, and 36 weeks postoperatively in the 36-week observation group rats (group A: *n* = 3, group B: *n* = 3).

### Estimation of metallic artifact

Both metallic clips formed metallic artifacts that interfered with image quality in CT. The artifacts’ appearances were evaluated on CT images.

### Estimation of degradation volume

The clips were extracted at the end of the observation period at 1, 4, 12, 24, and 36 weeks after surgery in group A (*n* = 15). To estimate the degradation rate, the remaining ratio, which was the volume after extraction divided by the volume before implantation, was calculated. The volume before implantation was estimated from the division of the weight by the alloy density. The volume after extraction was evaluated from the three-dimensional CT data obtained by micro-focus X-ray computerized tomography (μCT; ScanX-mate-E90, Comscantecno Co., Ltd., Yokohama, Japan).

### Histologic analysis

The tissue, including hepatocytes around clips, was extracted and fixed in 10% formalin, embedded in paraffin, and cut into 3-mm thick slices. Hematoxylin and eosin staining was performed to evaluate the degree of inflammatory reaction.

### Statistical analysis

Blood chemistry data were compared between the two groups using *t*-test. A *p* value < 0.05 was considered statistically significant. All calculations were performed with the help of JMP software version 10 (SAS Institute, Cary, NC, USA).

## Results

### Surgical outcomes

All rats survived surgery and no significant complications occurred throughout the observation period. No intra-abdominal hemorrhage was observed, and all clips were found in the implanted place and the vascular pedicle was closed on micro-CT images.

### Investigation of blood parameters

**Fig. 3:**
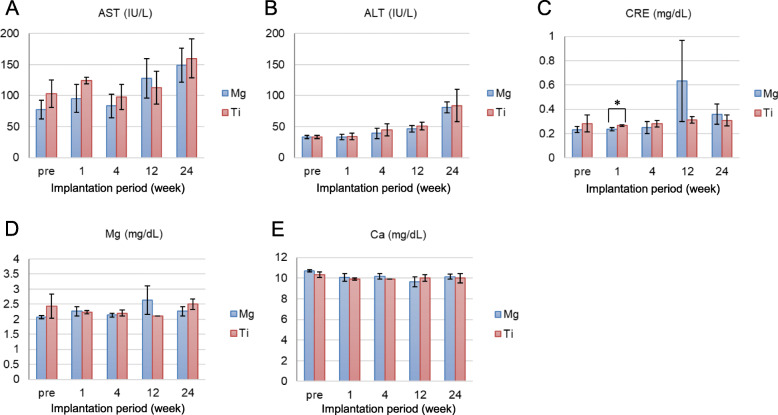
Blood biochemical values in the rats’ serum: (**a**) aspartate aminotransferase (AST), (**b**) alanine aminotransferase (ALT), (**c**) creatinine (Cre), (**d**) magnesium (Mg), and (**e**) calcium (Ca). ^*^There was a significant difference in the Cre level at 1 week after implantation

The blood test data of the 24-week observation group rats (group A: *n* = 3, group B: n = 3) are shown in Fig. 3. The serum AST and ALT levels gradually increased after surgery, but the AST and ALT levels were comparable between the two groups (Fig. 3a and b). In addition, there were no significant differences in the serum concentrations of Mg and Ca, which are components of the Mg alloy clips in both groups (Fig. 3d and e). There was a significant difference in the Cre level at 1 week after implantation (Fig. 3c).

### Micro-CT imaging data analysis

**Fig. 4:**
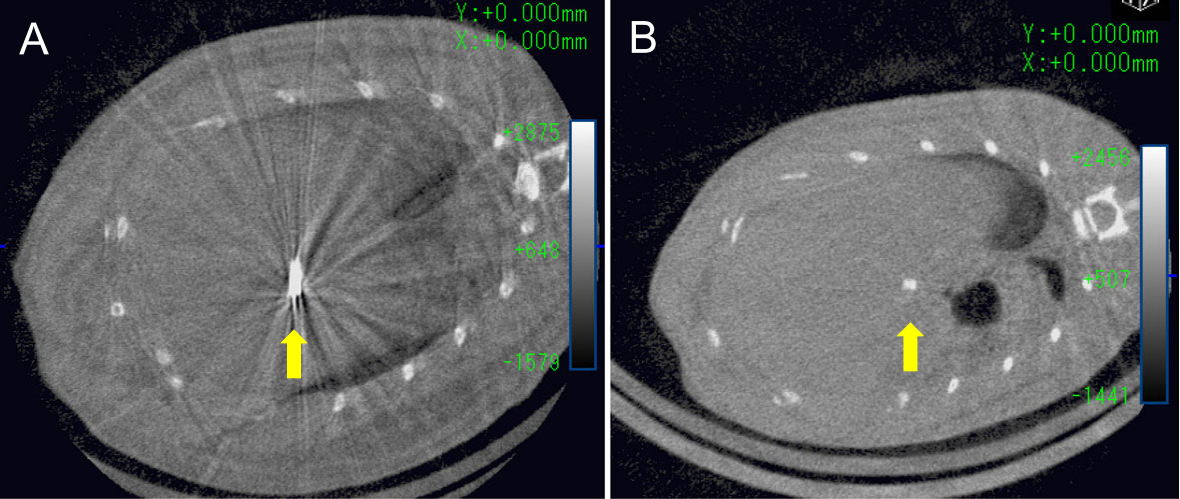
Computed tomography images just after hepatectomy. **a** Strong artifacts were formed radially from the titanium clip (yellow arrow). **b** Few artifacts were formed from the magnesium alloy clip (yellow arrow)

Figure 4 shows CT images of the rats in both groups just after surgery. Strong artifacts were formed radially from the Ti clips, as shown in Fig. 4a. Conversely, few artifacts were formed from the Mg alloy clips throughout the observation period, as shown in Fig. 4b.

### Ability for closure of the vascular pedicle

**Fig. 5:**
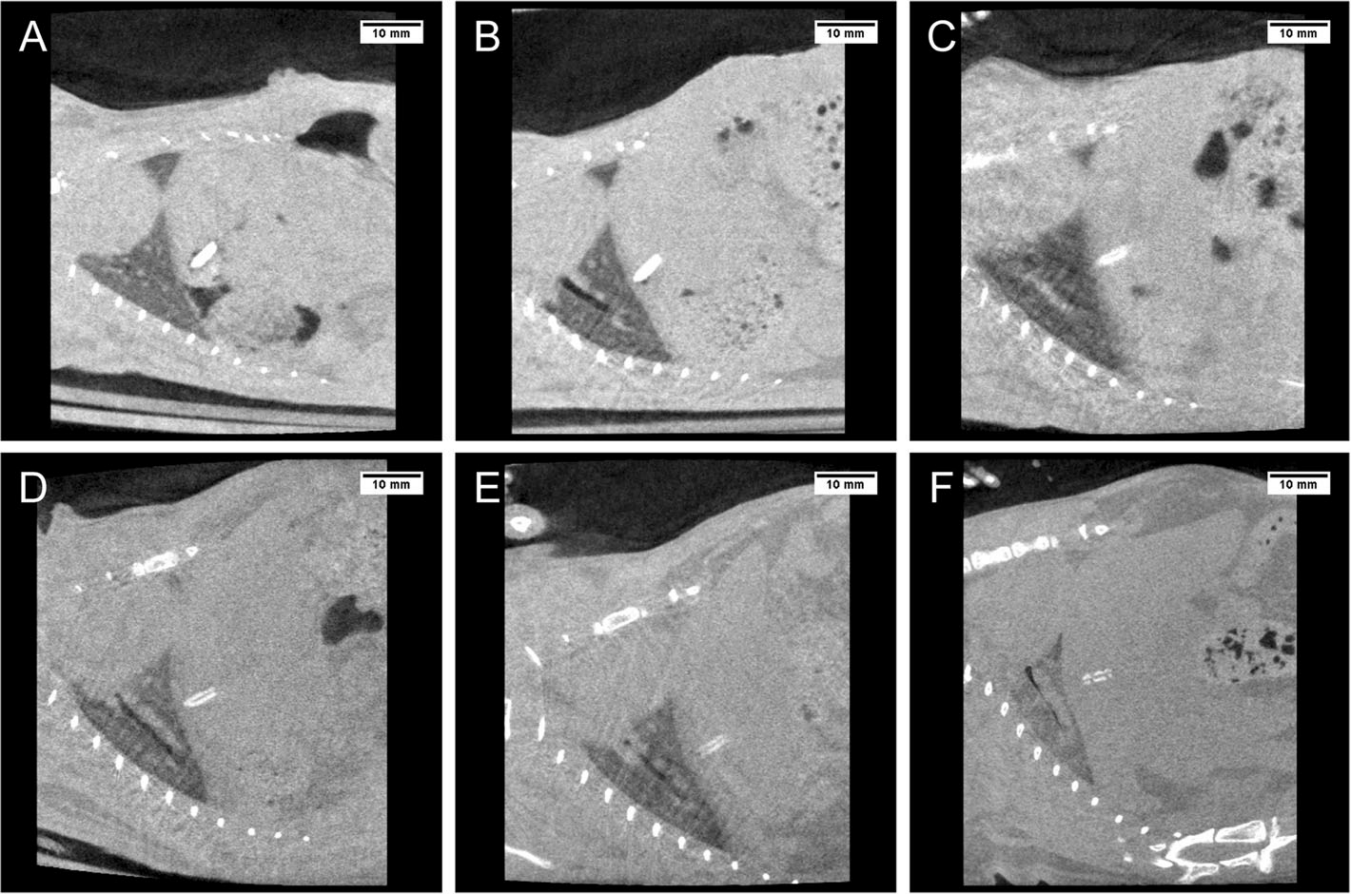
Computed tomography images of the same rat after hepatectomy using the magnesium alloy clip: (**a**) just after hepatectomy, (**b**) after 1 week, (**c**) after 4 weeks, (**d**) after 12 weeks, (**e**) after 24 weeks, and (**f**) after 36 weeks

Figure 5 shows the chronological change in the Mg alloy clips from just after surgery to 36 weeks in the 36-week observation group rats (group A: *n* = 3, group B: *n* = 3). The form of the Mg alloy clip was maintained within 4 weeks (Fig. 5a–c). Afterward, the Mg alloy clip became completely thin at 12 weeks (Fig. 5d) and split into two at 24 weeks (Fig. 5e). Finally, it was dismantled at 36 weeks (Fig. 5f). According to the CT images, the ability for closure of the vascular pedicle was confirmed until 12 weeks at least.

### Change in the volume of mg alloy clips

**Fig. 6:**
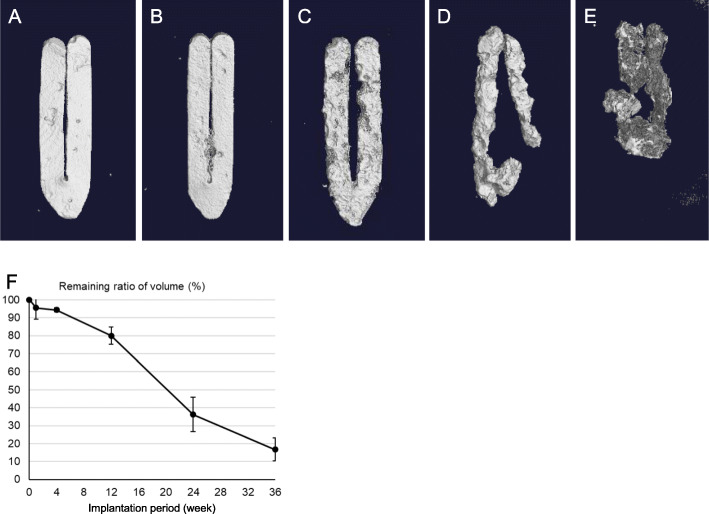
Three-dimensional computed tomography images of the magnesium alloy clip in the same rat after hepatectomy: (**a**) after 1 week, (**b**) after 4 weeks, (**c**) after 12 weeks, (**d**) after 24 weeks, and (**e**) after 36 weeks. The relationship between the remaining volume ratio and the implantation period is shown in (**f**)

The Mg alloy clips in 15 rats (group A: *n* = 15) were extracted and observed using micro-CT scanning to determine their degradation behavior. The 3D images reconstructed from the CT images are shown in Fig. 6a–e and correspond to 1, 4, 12, 24, and 36 weeks after surgery, respectively. The longer the clips were implanted, the thinner and smaller they became. Figure 6f shows that the calculated remaining volume ratio of the Mg alloy clip decreased gradually during the observation period. The remaining volume ratio of Mg alloy clips was 95.5, 94.3, 80.0, 36.2, and 16.7% at 1, 4, 12, 24, and 36 weeks, respectively.

### Histologic findings

**Fig. 7:**
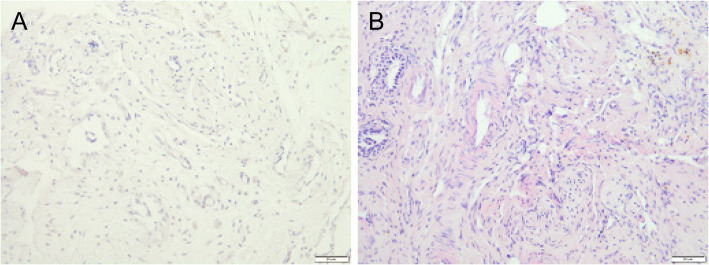
Hematoxylin and eosin staining of the tissue, including hepatocytes, around the clips at 12 weeks. **a** Titanium clip and (**b**) magnesium alloy clip. Scale bar: 20 μm

Hematoxylin and eosin staining of the tissue, including hepatocytes, around the clips at 12 weeks is shown in Fig. 7. Mild inflammation and fibrosis could be seen in the tissue around the clips in both groups (Fig. 7a and b). No significant difference was observed between both groups.

## Discussion

This study showed that our Mg alloy clips are feasible and sufficiently tolerable biodegradable surgical clips for vessel sealing during hepatectomy. In addition, there was no evidence that the serum concentration of Mg elevated throughout the observation period.

Our Mg alloy clips possessed sufficient properties for the 10-min occlusion of a renal vein in a rat model, and for the 24-week occlusion of a cystic duct in a canine model. [11, 12] However, the hemostatic performance and the degradation behavior of our Mg alloy clips after vessel sealing for a long observation period has not been evaluated yet. When we apply the Mg alloy clip for vessel sealing, we should consider the effects of the blood flow on the clip. After tightening the clip, it might cause absorption from the blood vessel wall or come in direct contact with the blood flow of capillary vessels. These conditions may induce rapid degradation of the clip, considerable hydrogen gas production, and elevation of serum Mg levels resulting in failure of vessel sealing. In this study, we first applied our Mg alloy clip for vessel sealing in rat hepatectomy and demonstrated that our Mg alloy clips possess sufficient characteristics for vessel sealing during hepatectomy.

Pure Mg has excellent biodegradability in a physiological environment. However, in turn, this high degradation rate of pure Mg and the resulting evolution of hydrogen gas are the main obstacles to clinical application as a surgical clip. To regulate the degradation velocity, several previous studies devised methods of coating using other materials such as gold, silicon, and polymer; these coating methods resulted in slow degradation. [14–18] However, the surface coating is in danger of peeling off through mechanical irritation during surgery or postoperatively. Moreover, it may trigger localized degradation through crevice corrosion. Therefore, we need to develop new ways to overcome this in vivo instability. Our Mg alloy clips showed continuous biodegradability in the abdominal cavity and approximately 64% of them were absorbed at 24 weeks (Fig. 6). This is slower than that seen in the canine cholecystectomy model; approximately 87% of the clips were absorbed at 24 weeks. This difference in biodegradability may be affected by the differences among animal species, targets of Mg alloy clips and circumstances of their placement. Altogether, we believe that this degradation speed is sufficient for vessel sealing because the clot formation in the vessel occurred early after tightening the clip.

Metallic artifacts that interfere with CT and magnetic resonance imaging are problematic. Modern CT units can reduce metallic artifacts induced by Ti or stainless steel by using advanced technologies. However, they result in reduced image quality [19] or high peak voltage of x-ray radiation. [20] Previous studies reported that replacing metal clips with polymeric absorbable clips reduce the artifacts. [21] However, currently available polymer clips have several disadvantages compared with metal clips. Since they are thicker and larger than metal clips, surgeons feel that it is difficult to close vessels without the sufficient margin of clipping. In addition, it is difficult to remove them because they have a rigid outer polyglycolic body or locking system. In contrast, our previous studies demonstrated that Mg alloy clips form fewer metallic artifacts than that with Ti clips. [12] Moreover, this study demonstrated that an Mg alloy clip maintains its shape for 12 weeks, the volume of the clip gradually decreases because of its biodegradability, and that metallic artifacts eventually completely disappear. Thus, visualization of ligation after using clips with minimal artifacts provides a sense of security when postoperative CT is performed to determine the cause of complications, at least in the perioperative period. Conversely, disappearance of the clips facilitates accurate diagnosis of recurrence of cancers without artifacts in the late postoperative phase.

In liver surgery, many vascular structures need to be ligated and divided during parenchymal transection. Over the last three decades, open and laparoscopic liver resection has been increasingly performed for the treatment of liver diseases, but the type of instruments by energy sources and technique used for hepatic transection vary among institutions. [22, 23] Although various vessel-sealing devices and ultrasonic scalpels have been developed and widely used, whether vascular systems, including bile ducts and thick vessels, are completely occluded with these devices has not been proven. Conversely, clip ligation of biliary and vascular structures is a safe and time-saving technique compared to ligation with thread. [13] Thus, we believe that Mg alloy clips are useful for ligation of biliary and vascular structures during hepatectomy. Additionally, surgeons who prefer metal clips to polymeric clips would believe that our Mg alloy clips close thin vessels relatively easily and remove themselves in mis-ligation cases similar to Ti clips.

The price of Mg alloys is an important issue. The market prices of raw Mg is approximately less than one third of that of Ti, and the melting temperatures of Ti and Mg are 1941 K and 923 K, respectively. These facts indicate that the fabrication cost of the present Mg-Zn-Ca alloy is considerably lower than that of conventional Ti alloy for medical application (e.g., Ti-6Al-4 V alloy). Therefore, we expect that the production cost of our Mg alloy clips would be lower than that of currently available Ti clips. Furthermore, the deformation behavior of our Mg alloy is relatively similar to that of Ti, compared with a polymer material, due to the work-hardening property and/or plastic deformation capability. This suggests that the Mg alloy clips can be used with conventional clip dispensers. We believe that this ability also contributes to the total production cost.

The main limitation of this study is that we only used a minimum number of healthy small animals for hepatectomy. Hepatectomy for patients with liver tumors requires much more ligation of vessels and leads to more intraoperative bleeding and postoperative ascites that might influence the degradation behavior of the Mg alloy clips. Thus, further examinations, including hepatectomy, in large animals are required to apply the Mg alloy clips in clinical situations.

In this study, we revealed that Mg alloy clips enabled closure of vessels with good hemostasis. In addition, there was no evidence that the serum Mg concentration elevated, and the degradation behavior was not problematic when we applied the biodegradable Mg alloy clips for vessel ligation in rat hepatectomy.

## Conclusions

This study aimed to examine the tolerability and safety of the novel biodegradable Mg alloy clips compared with those of standard Ti clips for hepatectomy in a rat model. These Mg alloy clips have the potential to replace standard metal clips in the near future. However, further studies are needed to investigate the use of biodegradable Mg alloy clips for the same indication in large animals as well as in clinical human models.

## Data Availability

The datasets generated and/or analyzed during the current study are available from the corresponding author on reasonable request.

## Abbreviations

ALT: Alanine aminotransferase
AST: Aspartate aminotransferase
Ca: calcium
Cre: creatinine
CT: Computed tomography
Mg: magnesium
MR: Magnetic resonance
Ti: titanium
Zn: zinc
